# SURMOUNT‐REAL UK: A Pragmatic Randomized Clinical Trial to Assess the Effectiveness of Tirzepatide in Adults With Obesity

**DOI:** 10.1002/oby.70247

**Published:** 2026-06-15

**Authors:** Martin K. Rutter, Julie Mount, J. Martin Gibson, Joanne Webb, Cheryl Marler, Catherine Reed, Mugdha Gore, Wenyu Ye, Elisa Gomez Valderas, John Wilding, Naveed Sattar, Melanie J. Davies, Kamlesh Khunti, Richard Haynes, Rachel L. Batterham

**Affiliations:** ^1^ Division of Diabetes, Endocrinology and Gastroenterology, School of Medical Sciences, Faculty of Biology, Medicine and Health University of Manchester Manchester UK; ^2^ Diabetes, Endocrinology and Metabolism Centre Manchester University NHS Foundation Trust, Manchester Academic Health Science Centre Manchester UK; ^3^ Eli Lilly and Company Basingstoke UK; ^4^ NorthWest EHealth (NWEH) Ltd. Manchester UK; ^5^ Eli Lilly and Company Indianapolis Indiana USA; ^6^ Department of Cardiovascular and Metabolic Medicine University of Liverpool Liverpool UK; ^7^ School of Cardiovascular and Metabolic Health University of Glasgow Glasgow UK; ^8^ Diabetes Research Centre University of Leicester Leicester UK; ^9^ NIHR Leicester Biomedical Research Centre Leicester UK; ^10^ Clinical Trial Service Unit and Epidemiological Studies Unit, Nuffield Department of Population Health University of Oxford Oxford UK

**Keywords:** electronic health records, obesity, pragmatic trial, primary care, type 2 diabetes incidence

## Abstract

**Objective:**

SURMOUNT‐REAL UK will evaluate the effectiveness of tirzepatide when offered in addition to standard‐of‐care (SoC) in adults with Class I obesity (BMI ≥ 30 and ≤ 34.9 kg/m^2^) and without diabetes in a UK primary care setting.

**Methods:**

A 5‐year, phase 4, multicenter, open‐label, pragmatic randomized clinical trial is enabled through access to participants' integrated electronic healthcare record data. The study will enroll approximately 3000 participants from Greater Manchester, UK, who are randomly assigned in a 1:1 ratio to receive either tirzepatide and SoC or SoC alone.

**Results:**

The primary endpoint is the percent change in body weight from baseline to Month 24, with the time to onset of type 2 diabetes to Month 60 being the key secondary endpoint. Additional endpoints include the impact of tirzepatide versus SoC on obesity‐related complications, health‐related quality of life, healthcare resource utilization, productivity, employment, and sickness‐related absences.

**Conclusions:**

SURMOUNT‐REAL UK employs a novel study design to evaluate real‐world health outcomes and potential long‐term benefits for both participants and the healthcare system associated with the delivery of pharmacological obesity treatment at a population level. The study is intended to generate critical evidence to support informed decision‐making in obesity management, clinical guideline development, and healthcare policy.

**Trial Registration:**

ClinicalTrials.gov identifier: NCT07247084

## Study Importance

- What is already known?
  ○ Obesity is a chronic, relapsing disease associated with significant morbidity and premature mortality. As the prevalence and extent of obesity increase, the population‐wide incidence of obesity‐related complications (ORCs) and the associated economic consequences also increase.
  ○ Tirzepatide is a glucose‐dependent insulinotropic polypeptide and glucagon‐like peptide‐1 receptor agonist indicated as an adjunct to a reduced‐calorie diet and increased physical activity for the treatment of adults with obesity or overweight in the presence of ≥ 1 weight‐related comorbid condition.
- What does this study add?
  ○ SURMOUNT‐REAL UK is a phase 4, pragmatic randomized clinical trial designed to extend the evidence generated by the SURMOUNT program. The study evaluates the effectiveness of tirzepatide on weight reduction and type 2 diabetes (T2D) risk when delivered in a UK primary care setting in adults living with Class I obesity (BMI ≥ 30 to ≤ 34.9 kg/m^2^).
  ○ In addition to data collected directly from participants, SURMOUNT‐REAL UK leverages linked electronic healthcare record data from primary and secondary care to evaluate population health and health system benefits of tirzepatide, including burden of multiple ORCs, healthcare resource utilization, and sickness‐related absences.
- How might these results change the direction of research or the focus of clinical practice?
  ○ In addition to examining weight loss and T2D prevention, the study will generate evidence of the broader patient, economic, and societal benefits of effective obesity treatment in routine clinical practice. The robust study design, generalizability, and health system‐relevant endpoints are intended to provide critical insights for obesity treatment decision‐making, clinical guidelines, and healthcare policy globally.

## 1 Introduction

Obesity is a chronic, relapsing, and commonly progressive disease if left untreated, which can result in a range of organ‐specific complications and premature mortality [1, 2, 3]. Obesity is a serious global health concern at both the individual and population level and it imposes a considerable economic burden due to higher healthcare resource utilization (HCRU) and lower work productivity, especially in cases of multimorbidity [4, 5, 6]. Obesity is associated with the development and progression of numerous obesity‐related complications (ORCs), including type 2 diabetes (T2D), and lower health‐related quality of life (HRQoL), and it can impair activities of daily living [7, 8, 9]. Modest and sustained weight loss can lead to clinically meaningful improvements in glycemic measures via reductions in ectopic fat, triglycerides, and other cardiometabolic parameters, all of which have been shown to reduce the risk of many ORCs, especially the development and progression of T2D [10]. Lifestyle‐based interventions for weight reduction form the foundation of obesity management; however, achieving and sustaining weight reduction can often be challenging, and weight regain can occur in the absence of treatment [11, 12, 13]. Incretin‐based obesity management medications (OMMs), such as glucagon‐like peptide‐1 (GLP‐1) receptor agonists and dual glucose‐dependent insulinotropic polypeptide (GIP) and GLP‐1 receptor agonists, are recommended treatments for obesity, as an adjunct to a reduced‐calorie diet and increased physical activity [13, 14, 15]. However, there is inequitable access to OMMs for people living with obesity in many countries and regions [16, 17]. Access barriers include a misalignment between the perception of obesity and its recognition as a disease, gaps in the evidence base of the long‐term effects of pharmaceutical interventions, challenges with obesity management policies, and concerns regarding affordability due to the large numbers of people living with obesity requiring healthcare services [16, 17, 18].

Tirzepatide is a once‐weekly injectable, subcutaneous dual GIP and GLP‐1 receptor agonist indicated for weight management, including weight loss and weight maintenance, as an adjunct to a reduced‐calorie diet and increased physical activity in adults with obesity (body mass index [BMI] ≥ 30 kg/m^2^) or overweight (BMI ≥ 27 to < 30 kg/m^2^) in the presence of ≥ 1 weight‐related comorbid condition [19, 20]. Tirzepatide has demonstrated benefits in weight management, T2D, and improving cardiometabolic health parameters and HRQoL; however, further evidence is needed to understand its impact in routine healthcare settings [21, 22, 23, 24, 25]. SURMOUNT‐REAL UK is a pragmatic randomized clinical trial (RCT) designed to provide critical insights into the value to both participants and the healthcare system of treatment of obesity at the population level. Tirzepatide will be used in this interventional study in accordance with the terms of its marketing authorization in the United Kingdom (UK), in participants with Class I obesity (BMI ≥ 30 and ≤ 34.9 kg/m^2^), to increase the evidence base for tirzepatide in this population and the rationale for treating obesity at an early stage in its natural history.

## 2 Methods

### 2.1 Study Design

SURMOUNT‐REAL UK is a 5‐year, phase 4, multicenter, open‐label, pragmatic RCT investigating the effectiveness of once‐weekly tirzepatide plus standard of care (SoC) compared to SoC alone in adult participants with Class I obesity (BMI ≥ 30 and ≤ 34.9 kg/m^2^) and without diabetes in UK primary care. The SURMOUNT‐REAL UK study conforms to the Declaration of Helsinki. The study protocol was approved by Wales REC 5 Research Ethics Committee (25/WA/0237), and all patients are required to provide written, informed consent before participating in the study.

The study will be nested within primary care physician healthcare networks that have routine participant‐level electronic health record (EHR) coverage from primary care, which can be integrated with secondary care services. The use of integrated EHR enables the long‐term evaluation of ORCs, HCRU, and sickness‐related absence through routine passive data collection, alongside active study assessments of weight, cardiometabolic parameters, HRQoL, work productivity, and employment status. Oversight of the study participants, including prescribing decisions, will be provided by local primary care teams throughout the trial. During the treatment initiation period, interactions with study participants will be primarily conducted via telehealth visits, which will occur every 4 weeks for the first 20 weeks, with participant‐initiated visits occurring as needed through Month 12. After Month 12, telehealth visits will occur at 6‐month intervals, with participant‐initiated visits occurring as needed throughout the trial. Study‐specific site visits are intentionally limited and occur only at 12, 24, and 60 Months post screening. The main features of the study design are shown in Figure 1.

**FIGURE 1:**
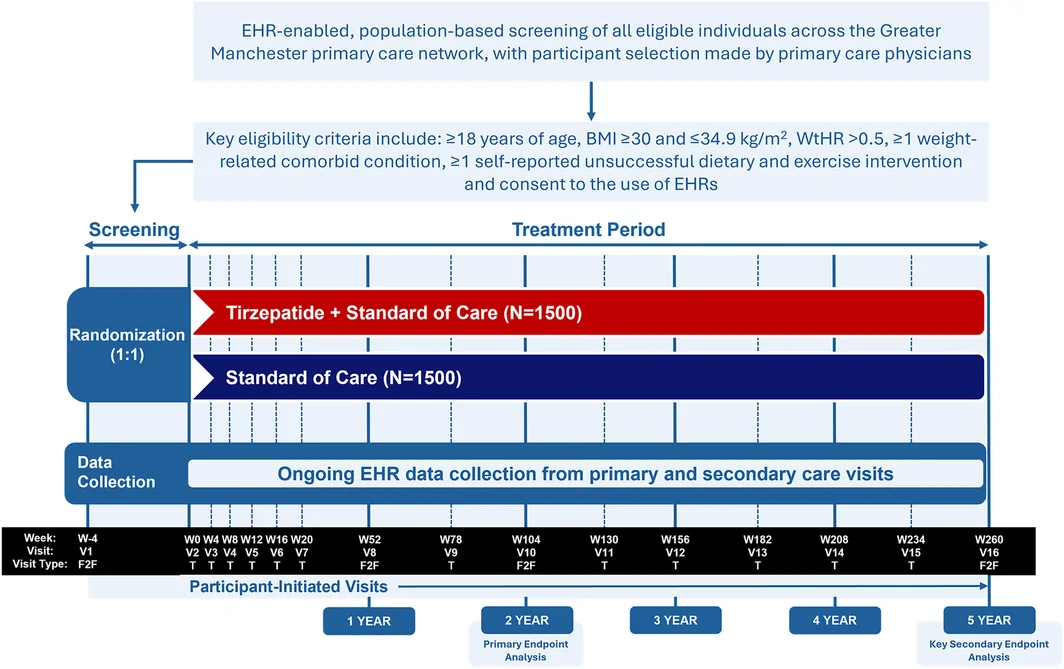
SURMOUNT‐REAL UK study schema. EHR = electronic health record; F2F = face‐to‐face; T = telehealth; V = Visit; W = Week; WtHR = waist to height ratio.

### 2.2 Study Procedures and Participants

SURMOUNT‐REAL UK will enroll approximately 3000 adult participants with Class I obesity, randomly assigned in a 1:1 ratio to receive either tirzepatide and SoC or SoC alone. Participants who meet all criteria for enrolment will be randomly assigned using an interactive web‐response system and stratified based on sex and prediabetes status (HbA1c < 42 mmol [6.0%], HbA1c ≥ 42 mmol [6.0%]) based on Diabetes UK guidance. The randomization sequence is not known to personnel responsible for enrolling participants. The expected total duration of participation, including screening (28 days) and treatment, is approximately 5 years. Trial sites will be local primary care clinics in Greater Manchester, UK, with each site led by a single primary care physician who will serve as a Principal Investigator (PI). To enhance generalizability, all potentially eligible participants will be identified from population‐based EHR screening using the FARSITE tool, with the PI making the final selection of suitable participants for entry into the trial [26]. A central study research team will provide centralized safety monitoring, oversight of the study, and support to the primary care team to minimize administrative requirements. Participants may be eligible for this study if they:

- are ≥ 18 years of age,
- have BMI ≥ 30 and ≤ 34.9 kg/m^2^ (Class I obesity, adjusted for ethnicity [BMI threshold reduced by 2.5 kg/m^2^ for people from South Asian, Chinese, other Asian, Middle Eastern, Black African, or African‐Caribbean ethnic backgrounds]),
- have an increased waist to height ratio (defined by > 0.5),
- have ≥ 1 weight‐related comorbid condition (e.g., cardiovascular conditions, metabolic disorders, other obesity‐related conditions), and
- have a history of ≥ 1 self‐reported unsuccessful dietary and exercise intervention aimed at reducing body weight (Table 1).

**TABLE 1:** Inclusion and exclusion criteria.

Inclusion criteria
**Age, type of participant, and disease characteristics** ≥ 18 years of age BMI ≥ 30 and ≤ 34.9 kg/m^2^ *Note:* BMI threshold reduced by 2.5 kg/m^2^ for people from South Asian, Chinese, other Asian, Middle Eastern, Black African, or African‐Caribbean ethnic backgrounds Increased waist to height ratio (defined by > 0.5) ≥ 1 weight‐related comorbid condition: Cardiovascular conditions: Hypertension Dyslipidemia Heart failure Pulmonary artery hypertension Atrial fibrillation Atherosclerotic cardiovascular disease, including ischemic heart disease (such as coronary artery disease and angina), ischemic stroke, and/or transient ischemic attack Metabolic disorders: Current prediabetes (non‐diabetic hyperglycemia) Other obesity‐related conditions: Obstructive sleep apnea Metabolic–associated fatty liver disease or metabolic dysfunction–associated steatohepatitis Polycystic ovary syndrome Osteoarthritis of the hip or knee Microalbuminuria with reduced eGFR History of ≥ 1 unsuccessful self‐reported effort to reduce body weight based on diet and exercise Individuals of childbearing potential (not surgically sterilized and between menarche and 1 year post menopause) who are randomized to the tirzepatide arm must: test negative for pregnancy at Visit 1 based on a serum pregnancy test, if sexually active, agree to use an effective form of contraception, if using an oral form of contraceptive, agree to switch to a non‐oral contraceptive method or add a barrier method of contraception upon initiating tirzepatide therapy (for 4 weeks) and after each dose escalation (for 4 weeks) not be breastfeeding **Informed consent** 7 Capable of giving signed informed consent, which includes compliance with the requirements and restrictions listed in the informed consent form and study protocol **Other inclusion criteria** 8 Consent to the use of electronic health records for research purposes 9 In the investigator's opinion, participants are well‐motivated, capable, and willing to learn how to self‐inject the study intervention, as required for this study **Exceptions:** Visually impaired participants who are not able to self‐administer the injections must have the assistance of a sighted individual trained to inject the study intervention Participants with physical limitations who are not able to self‐administer the injections must have the assistance of an individual trained to inject the study intervention Inject study intervention (or receive an injection from a trained individual if visually impaired or with physical limitations)

Exclusion criteria
**Medical Conditions** *Diabetes related* T1D, T2D, or any other type of diabetes (except prior gestational diabetes), history of ketoacidosis, or hyperosmolar state/coma Laboratory evidence diagnostic of diabetes at screening, indicated by HbA1c ≥ 6.5% (≥ 48 mmol/mol) *Obesity related* 3 Self‐reported or documented change in body weight > 5 kg within 90 days prior to screening 4 Prior or planned surgical treatment for obesity **Exceptions**: The following are allowed if they occurred > 1 year before screening: liposuction, cryolipolysis, or abdominoplasty 5 Prior or planned endoscopic (e.g., mucosal ablation, gastric artery embolization) and/or device‐based (e.g., lap band, intragastric balloon, duodena‐jejunal endoluminal liner) therapy for obesity *Note:* Prior device‐based therapy is acceptable if device removal was > 6 months prior to screening *Hematologic* 6 Any hematological condition that may interfere with HbA1c measurement (e.g., hemolytic anemias, sickle cell disease) *Malignancy* 7 History of an active or untreated malignancy or are in remission from a malignancy for < 5 years 8 Family (first‐degree relative) or personal history of medullary thyroid carcinoma or multiple endocrine neoplasia syndrome type 2 *Other medical conditions*: 9 Known history of severe gastrointestinal disease (e.g., severe gastroparesis, gastric outlet obstruction) 10 History of chronic or acute pancreatitis 11 Obesity induced by other endocrinologic disorders (e.g., Cushing syndrome) or diagnosed monogenetic or syndromic forms of obesity (e.g., melanocortin 4 receptor deficiency, Prader Willi syndrome). 12 History of significant active or unstable major depressive disorder (MDD) or other severe psychiatric disorder (e.g., schizophrenia, bipolar disorder, other serious mood or anxiety disorder) within the last 2 years that in the opinion of the investigator poses an unacceptable risk if participating in the study or of interfering with the interpretation of data *Note:* Participants with MDD or generalized anxiety disorder whose disease state is considered stable for the past 2 years and expected to remain stable throughout the course of the study, in the opinion of the investigator, may be considered for inclusion 13 Known allergies or intolerance to GLP‐1 receptor agonists, GIP and GLP‐1 receptor agonists, or excipients **Prior/concomitant therapy** 14 Received any incretin therapy within the past 12 months (GLP‐1 receptor agonists or GIP and GLP‐1 medications) **Prior/concurrent clinical study experience** 15 Currently enrolled in any other clinical study involving an investigational product (IP) or any other type of medical research judged not to be scientifically or medically compatible with this study 16 Within the last 30 days, participated in a clinical study and received treatment, whether active or placebo; if the study involved an IP, 5 half‐lives or 30 days, whichever is longer, should have passed 17 Previously completed or withdrawn from this study or any other study investigating tirzepatide after receiving at least 1 dose of IP **Other exclusion criteria** 18 Are planning to move out of the geographical area covered by the study's EHR network at any point during the duration of the study 19 Are investigator site personnel directly affiliated with this study or their immediate family; immediate family is defined as a spouse, parent, child, or sibling, whether biological or legally adopted 20 Are Lilly employees, or are employees of any third party involved in the study, who require exclusion of their employees

Abbreviations: eGFR = estimated glomerular filtration rate; EHR = electronic healthcare record; GIP = glucose‐dependent insulinotropic polypeptide; GLP‐1 = glucagon‐like peptide‐1; HbA1c = hemoglobin A1c; T1D = type 1 diabetes; T2D = type 2 diabetes.

To participate in the study, participants must also be capable of giving informed consent, which includes consent to the use of their EHR for research purposes.

Key exclusion criteria include a preexisting diagnosis of diabetes or laboratory evidence suggestive of diabetes at screening, a self‐reported change in body weight > 5 kg within 90 days prior to screening, prior or planned surgical treatment for obesity, prior or planned endoscopic or device‐based therapy for obesity, or any incretin therapy ≤ 12 months before screening. Participants must also intend to remain within the geographical area covered by the EHR network for the duration of the trial. A complete list of eligibility criteria is available in Table 1.

### 2.3 Intervention

Participants will be offered either tirzepatide and SoC or SoC alone. The starting dose of tirzepatide will be 2.5 mg, administered subcutaneously once weekly. After 4 weeks, participants will be advised to increase the dose to 5 mg once weekly. If needed, dose increases will be made in 2.5 mg increments after a minimum of 4 weeks on the current dose. The recommended maintenance doses are 5, 10, and 15 mg. The maximum dose will be 15 mg once weekly. Dose escalation/de‐escalation, or the speed of escalation, will follow a shared decision‐making model between the participant and physician as employed in routine clinical practice based on individual participant needs, tolerability, and response. Participants in the SoC arm will not receive placebo treatment in this pragmatic, open‐label trial.

All study participants will be provided with dietary and physical activity advice and will be encouraged to engage with dietary or lifestyle management counseling throughout the study as part of SoC, including National Health Service (NHS) SoC obesity services, which may include, but are not limited to:

- Counseling on diet, physical activity, and behaviors, using overweight and obesity management pathways and local services
- NHS Better Health website and apps
- NHS Digital Weight Management Programme
- NHS Diabetes Prevention Programme, for individuals with non‐diabetic hyperglycemia (prediabetes), and
- NHS Type 2 diabetes Path to Remission Programme, for individuals who develop T2D during the trial

Availability and eligibility for SoC services will be in accordance with resources provided within routine care. Services may vary in intensity and treatment duration, and it is anticipated that the standard weight management support available within the national healthcare system may change throughout the course of the study, as well as participant eligibility for services.

Study participants will be permitted to use concomitant medications as needed for the duration of the study, regardless of treatment arm, inclusive of OMMs by SoC participants. Any study participant from the SoC treatment arm who develops T2D will remain in the study for the duration of the treatment period. The decision to further evaluate incident diabetes or initiate antihyperglycemic therapy and the choice of antihyperglycemic therapy, including the use of tirzepatide, will be at the discretion of the participant's primary care physician.

### 2.4 Objectives and Endpoints

The primary objective of SURMOUNT‐REAL UK is to demonstrate that tirzepatide and SoC are superior to SoC alone for change in body weight among participants with obesity and without diabetes. The primary endpoint of this study will be the percent change in body weight from baseline to Month 24. The key secondary objective and endpoint of this study are to demonstrate that tirzepatide and SoC are superior to SoC for delayed progression to T2D. The key secondary endpoint will be the time to onset of T2D from baseline to Month 60. In addition to the key study objectives, other objectives include comparing participants randomized to tirzepatide and SoC to SoC alone for change in various parameters such as glycemic status, body composition, lipid and liver laboratory parameters, blood pressure, and kidney function. Lipids and blood pressure are important obesity‐related cardiometabolic risk factors and will be assessed to characterize the broader effects of treatment with tirzepatide versus SoC alone. Intervention‐related differences in HCRU, such as primary care, specialist, and emergency room visits and hospital admissions, the percentage of patients who develop new ORCs, such as prediabetes and T2D, and patient‐reported outcomes (PROs) measuring HRQoL will also be evaluated. The study's exploratory objectives examine real‐world utilization of tirzepatide and intervention‐related differences in general medication burden, PROs for work productivity and activity impairment, and impact on employment status and sickness‐related absence. A list of key study objectives is available in Table 2.

**TABLE 2:** Key study objectives.

Primary	Percent change from baseline in body weight to Month 24
Key secondary	Time to onset of T2D from baseline to Month 60
Additional secondary	**Change from baseline in:** Body weight Body composition ⚬ Waist circumference ⚬ WtHR ⚬ BIA Glycemic status/control ⚬ Percentage of participants converting from prediabetes to normoglycemia ⚬ Percentage of participants with normoglycemia at baseline who develop prediabetes ⚬ Change in HbA1c Lipid parameters ⚬ Total cholesterol ⚬ Non‐HDL cholesterol ⚬ Triglycerides Liver function ⚬ ALT ⚬ AST Blood pressure ⚬ SBP ⚬ DBP Kidney function ⚬ UACR HRQoL ⚬ EQ‐5D‐5L ⚬ IWQOL‐Lite‐Clinical Trials HCRU ⚬ Primary care ⚬ Specialist outpatient ⚬ Emergency room ⚬ Hospital admission Incidence of study‐defined ORCs
Exploratory	Change in prevalent ORCs/existing conditions Change in mental health status ⚬ EQ‐5D‐5L ⚬ IWQOL‐Lite‐Clinical Trials PRO measures for productivity ⚬ WPAI Sickness‐related absence for employed participants Employment status Change in medications Tirzepatide utilization and adherence

Abbreviations: ALT = alanine aminotransferase; AST = aspartate aminotransferase; BIA = bioelectric impedance analysis; DBP = diastolic blood pressure; HbA1c = hemoglobin A1c; HCRU = healthcare resource utilization; HRQoL = health‐related quality of life; ORC = obesity‐related complication; PRO = patient‐reported outcome; SBP = systolic blood pressure; T2D = type 2 diabetes; UACR = urinary albumin to creatinine ratio; WPAI = Work Productivity and Activity Impairment; WtHR = waist to height ratio.

### 2.5 Discontinuation

When necessary, a participant may be discontinued from the study intervention. If the study intervention is discontinued, participants will be encouraged to remain in the study and attend all scheduled study visits to enable assessment of study objectives. Temporary dose interruptions of the study intervention are permitted throughout the study. The decision and duration of any temporary interruption of study intervention will also follow a shared decision‐making model and be at the discretion of both the investigator and participant. A participant can also withdraw from study procedures, whether randomized to the tirzepatide and SoC arm or the SoC alone arm; unless permission is withdrawn, capture of the data through the EHR will continue reducing the impact of missing data. For participants in the tirzepatide and SoC arm who withdraw from the study, study provision of tirzepatide will be permanently discontinued. Any participant who discontinues before the last visit in the treatment period will have an early discontinuation visit to complete the study procedures. Participants will be considered lost to follow‐up if they repeatedly fail to return for scheduled visits, if they are unable to be contacted by the study site, and/or if access to EHR data is no longer available.

### 2.6 Assessments

Study procedures will be performed during screening and at Months 12, 24, and 60. Study‐specific procedures and assessments are intentionally limited to reduce the burden on participants and to maintain the experience of usual primary care delivery. Procedures will include physical assessments (height, body weight and composition, and blood pressure), collection of PRO measures, and laboratory samples. Physical assessments will be guided by the WHO's standardized physical measurement protocols for the STEPwise approach to surveillance [27]. Participants will be excluded from bioelectric impedance analysis if they have electronic implants, an active prosthesis to the arms or legs, or a connected device. For participants who are pregnant or have cardiac arrhythmia, bioelectric impedance analysis can only be performed with the consent of their PI. Assessments will be performed either at face‐to‐face (F2F) study site visits, telehealth visits, or passively via the participants' EHR. EHR data will be integrated with primary data collected from F2F, pharmacy, and telehealth visits using an electronic data capture (EDC) system. This hybrid data‐capture method enables assessments to be completed using EDC, EHR, or a combined approach. Assessments related to the primary objective will be performed during F2F study site visits, and body weight measurements will be recorded using the EDC system. Assessments for the key secondary objective will be performed during F2F visits in addition to passive EHR data collection. For the key secondary objective, incident T2D will be assessed by either a HbA1c value of at least 6.5% (48 mmol/mol) based on study EDC laboratory assessments or EHR data or a documented clinical diagnosis code of T2D in the participants' EHR. PROs will be collected via EDC at F2F visits and will include IWQOL‐Lite‐Clinical Trials, EQ‐5D‐5L, and Work Productivity and Activity Impairment (WPAI). Adverse events, serious adverse events, and product complaints (PCs) will be collected throughout the trial at study visits, when reported by the participant during routine care visits or by study staff, or identified through the EHR.

### 2.7 Statistical Considerations

A total of 3000 participants will provide over 95% statistical power to demonstrate the superiority of tirzepatide and SoC versus SoC alone for the primary endpoint on the mean percent change in body weight from baseline to Month 24 at a two‐sided significance level of 0.05. This sample size calculation assumes at least a 5% difference in mean percent weight change from baseline to Month 24 for tirzepatide and SoC as compared with SoC alone and a common standard deviation of 14%. This sample size is also expected to provide over 80% power to show superiority of tirzepatide and SoC versus SoC alone for the risk of developing T2D from baseline to Month 60, which is the key secondary endpoint. This power calculation is based on the following assumptions: a 0.3 hazard ratio and a two‐sided significance level of 0.05. Additional assumptions for both the primary and key secondary endpoints include a 26% annual treatment discontinuation rate and annual study discontinuation rates of 13% in the tirzepatide and SoC arm and up to 21% in the SoC alone arm.

All effectiveness and safety analyses will use the intention‐to‐treat (ITT) population, which includes all randomized participants. Two modified treatment‐regimen estimands and one efficacy estimand will be used in the analysis of this novel trial, with strategies employed to account for the potential for intercurrent events, including, but not limited to, discontinuation of tirzepatide in the tirzepatide and SoC arm or initiation of OMM treatments in the SoC alone arm. For the modified treatment‐regimen estimands, a hybrid approach incorporating both treatment‐policy and hypothetical strategies will be used to address intercurrent events. For the efficacy estimand, a hypothetical strategy assuming continued adherence to the randomized treatment will be applied. The primary estimand for the primary and key secondary objectives of this study is one of the modified treatment‐regimen estimands, which aims to demonstrate that tirzepatide plus SoC is superior to SoC alone in reducing body weight from baseline to Month 24 or prevention of T2D up to Month 60. The primary endpoint, percent change in body weight from baseline to Month 24, will be estimated using modified treatment‐regimen estimands via an ANCOVA model and an efficacy estimand via a mixed model for repeated measures. For ANCOVA, missing Month 24 body weight will be imputed based on the reason for missingness. For the key secondary endpoint, the time to onset of T2D will be analyzed using a Cox proportional hazards model. Participants who do not develop T2D by the end of follow‐up will be censored at the end of their follow‐up period, which will vary depending on the estimands. Missing data due to censoring will be addressed using the Cox model, assuming that censoring is independent of the outcome.

The familywise type I error for the primary and key secondary endpoints will be controlled using a gatekeeping strategy [28]. The time to onset of T2D will be assessed for statistical significance only if the primary endpoint is also significant. No multiplicity adjustments will be applied to other endpoint analyses. Additional exploratory analyses, as well as safety, subgroup, and interim analyses, may be conducted based on participant enrollment and follow‐up duration.

## 3 Discussion

SURMOUNT‐REAL UK is expected to provide a comprehensive assessment of the effects of tirzepatide on body weight and risk of T2D when delivered through usual primary care pathways, thereby complementing the evidence generated by the phase 3 SURMOUNT trials. The trial also addresses a substantial unmet need for long‐term, real‐world data on obesity interventions across outcomes of importance to patients, healthcare providers, and policy makers, including impacts on multiple ORCs, HCRU, medication burden, productivity, employment, and sickness‐related absence.

The study design of SURMOUNT‐REAL UK features various noteworthy aspects. Firstly, the study will be conducted within the UK, which was strategically selected because of its scientific and operational advantages. In the UK, healthcare is provided through the National Health Service (NHS), which is free at the point of care. Primary care providers serve as the main access point for healthcare services. The trial will be conducted in Greater Manchester and nested across regional primary care networks using the primary care EHR linked to secondary care data. This infrastructure is crucial in supporting the recruitment of a diverse study population, as well as in collecting ORCs outcome data. Access to the trial will also be enhanced with the use of mobile health units which participants can attend in local settings for study assessments, reducing the burden of travel. Greater Manchester is a region in the UK with a population of approximately 3 million, with a diverse socioeconomic and ethnic profile. In 2024, more than 1 in 4 adults in Greater Manchester were living with obesity, which was higher than the national average [29]. Greater Manchester has also supported other real‐world EHR‐enabled trials, demonstrating the region's ability to deliver SURMOUNT‐REAL UK [30, 31]. Collectively, these factors support the selection of this region as an optimal setting for this pragmatic study design, enhancing the validity and generalizability of study outcomes.

This trial will be conducted using an EDC platform to integrate data from scheduled study visits and assessments designed to standardize capture of key trial endpoints with routinely collected point‐of‐care data from primary care, secondary care, and pharmacies. The use of the EHR permits trial assessments to be kept to a minimum, which should reduce participant burden, improve retention, and preserve the pragmatic nature of the study. Primary care records will provide information about participants' health, medical history, treatments, and interactions with the healthcare system. The secondary care dataset will use Hospital Episode Statistics, which captures details of all admissions, outpatient appointments, and emergency department attendances at NHS hospitals. Prescription data will enable the assessment of prescribing trends, medication burden, and the real‐world adherence, persistence, and utilization of tirzepatide. EHR data will form the backbone of the study infrastructure and will be used to capture baseline and endpoint data, as well as to enable continuous, passive, and real‐time surveillance of study safety events. In the event that a participant withdraws from study procedures, data capture via the EHR will remain possible if consent is not withdrawn, thereby minimizing the impact of missing data.

The study is designed to reflect the impact of making tirzepatide available at no financial cost to people living with Class I obesity, an elevated waist to height ratio, and at least one weight‐related comorbidity in primary care. To capture the full real‐world experience of participants, the discontinuation (permanent or temporary) of tirzepatide in the tirzepatide and SoC arm, or the use of OMMs in the SoC arm, will not result in the participant's withdrawal from the study and prescribing and dosing decisions will be at the discretion of the prescribing PI and the participant. This approach ensures that the analysis more accurately captures the impact of actual treatment decisions and reflects the clinical realities of a noncontrolled setting.

The inclusion of participants with Class I obesity (BMI ≥ 30 and ≤ 34.9 kg/m^2^), waist to height ratio > 0.5, and ≥ 1 weight‐related comorbidity will contribute to the existing evidence base for tirzepatide in this BMI category. This may further support the rationale for earlier intervention in obesity care, which could help reduce both weight and T2D risk, as well as the risks of other long‐term ORCs, HCRU, sickness‐related absence, and loss of work productivity. Tirzepatide will be used in this interventional study in accordance with its marketing authorization in the UK. This means that tirzepatide is licensed for use as an adjunct to a reduced‐calorie diet and increased physical activity, the combination of which is essential for sustained health outcomes.

The use of new eligibility criteria, such as the requirement of an increased waist to height ratio (defined as ≥ 0.5), emphasizes the potential future relevance of these criteria for using OMMs in obesity management [2, 3]. Additional new elements include the usual care setting and long study duration, which, when combined, facilitate the long‐term evaluation of tirzepatide's effectiveness, safety, tolerability, adherence, and sustainability of weight loss, mirroring real‐world treatment patterns and outcomes over an extended period.

Incident T2D, percent change in body weight from baseline to Month 60, occurrence of new ORCs, HCRU, and sickness‐related absence are important endpoints of this trial reflecting overall participant health, work productivity, and HRQoL, respectively. In particular, the prevention of new‐onset T2D is a key secondary endpoint of this study, given its public health importance including the long‐term prevention of diabetes‐related complications [32]. The percent change in body weight from baseline to Month 60 is an important secondary endpoint for this study, enabling the assessment of long‐term continued treatment on weight reduction. Additionally, questionnaires that measure HRQoL and work productivity are also important tools that can capture participants' perspectives on their health and potentially help to inform decisions related to the treatment of obesity in primary care. In particular, the WPAI instrument, which assesses the effect of a target health problem on absenteeism, presenteeism, work productivity, and activity impairment, will support the assessment of employment status and sickness‐related absence data collected via the participants' EHR [33]. Furthermore, the questionnaires measuring physical and psychosocial health domains will provide insight into the participants' overall health perception throughout the study. Evidence on patient HRQoL is important to help demonstrate the effectiveness of tirzepatide for improving HRQoL and work productivity in a usual care setting, given that real‐world factors have the potential to influence participants' day‐to‐day functioning.

The SURMOUNT‐REAL UK study design has limitations. The open‐label study design, including the lack of placebo to blind investigators and participants to arm assignment, has the potential to contribute to ascertainment bias through differential loss to follow‐up. Furthermore, although participant recruitment is supported by the EHR and designed to be inclusive, differential uptake across sociodemographic groups may result in a trial population that is not fully representative of individuals with obesity. To reduce bias and strengthen the prespecified analyses, the statistical analysis plan was developed and approved before the first patient visit. Despite these limitations, the study has been rigorously designed and is expected to provide robust, actionable evidence for healthcare policy focused on obesity management and intervention in addition to diabetes prevention.

## 4 Conclusion

SURMOUNT‐REAL UK will evaluate the long‐term health outcomes and potential population health benefits of tirzepatide for people living with obesity and ≥ 1 weight‐related comorbid condition, but without diabetes. This trial aims to bridge the gap between the proven efficacy of tirzepatide in RCTs and its long‐term real‐world effectiveness in routine clinical practice. The robust study design, generalizability, and health system‐relevant endpoints are intended to provide critical insights for obesity treatment decision‐making, clinical guidelines, and healthcare policy.

## Funding

Funding for the SURMOUNT‐REAL UK trial was provided by Eli Lilly and Company.

## Conflicts of Interest

Julie Mount, Mugdha Gore, Elisa Gomez Valderas, Wenyu Ye, Cheryl Marler, Joanne Webb, Catherine Reed, and Rachel L. Batterham are employees of and shareholders in Eli Lilly and Company. Martin K. Rutter received consulting fees from Eli Lilly and Company and reports modest stock ownership in GSK. J. Martin Gibson reports serving as the chief medical officer on the executive board of NorthWest EHealth Ltd. and that NorthWest EHealth Ltd. received payments from Eli Lilly and Company. Richard Haynes received grants/contracts from Boehringer Ingelheim and reports participation on a data monitoring committee for Eli Lilly and Company; the Nuffield Department of Population Health at the University of Oxford has a staff policy of not accepting honoraria or consultancy fees directly or indirectly from industry. Kamlesh Khunti received grants/contracts from AstraZeneca, Boehringer Ingelheim, Eli Lilly and Company, Merck Sharp & Dohme, Sanofi, Servier, Oramed Pharmaceuticals, Roche, Daiichi‐Sankyo, and Applied Therapeutics, consulting fees from Amgen, AstraZeneca, Bristol Myers Squibb, Boehringer Ingelheim, Eli Lilly and Company, Novo Nordisk, Sanofi, Servier, Roche, Daiichi‐Sankyo, Embecta, and Nestle Health Science, and payment or honoraria for lectures, presentations, or speakers bureaus from Amgen, AstraZeneca, Bristol Myers Squibb, Boehringer Ingelheim, Eli Lilly and Company, Novo Nordisk, Sanofi, Servier, Roche, Daiichi‐Sankyo, Embecta, and Nestle Health Science. John Wilding received consulting fees from Alnylam, Amgen, AstraZeneca, Boehringer Ingelheim, Cytoki, Kailera, Eli Lilly and Company, Menarini, Metsera, Napp, Novo Nordisk, Pfizer, Prosciento, Response Pharmaceuticals, Rhythm Pharmaceuticals, Saniona, Shionogi, and Ysopia, payment or honoraria for lectures, presentations, or speakers bureaus from AstraZeneca, Boehringer Ingelheim, Medscape, Menarini, and Novo Nordisk, and support for attending meetings/travel from Menarini, Novo Nordisk, and Eli Lilly and Company and reports a leadership or fiduciary role for Diabetes Research and Wellness Foundation Grants Board, Rank Prize Funds Nutrition Committee, NIHR Metabolic and Endocrine Group, and World Obesity Federation (2018–2024). Melanie J. Davies received grants/contracts from AstraZeneca, Boehringer Ingelheim, and Novo Nordisk, consulting fees from Boehringer Ingelheim, Eli Lilly and Company, Novo Nordisk, Sanofi, and Kailera, payment or honoraria for lectures, presentations, or speakers bureaus from AstraZeneca, Boehringer Ingelheim, Novo Nordisk, Sanofi, Eli Lilly and Company, and Zuellig Pharma, and support for attending meetings/travel from Boehringer Ingelheim, Novo Nordisk, Eli Lilly and Company, Amgen, AstraZeneca, Biomea Fusion, Regeneron, and Zealand Pharma and reports participation on a data safety monitoring board or advisor board for Amgen, AstraZeneca, Biomea Fusion, Sanofi, Zealand Pharma, Carmot/Roche, Regeneron, EktaH, AbbVie, GSK, Daewoong Pharmaceuticals, and Innovent Bio. Naveed Sattar received grants/contracts from AstraZeneca, Boehringer Ingelheim, Novartis, and Roche; consulting fees from AbbVie, Amgen, AstraZeneca, Boehringer Ingelheim, Carmot Therapeutics, Eli Lilly and Company, Gan & Lee, GlaxoSmithKline, Hanmi Pharmaceuticals, Kailera, Mass Medicines, Menarini‐Ricerche, Metsera, Novo Nordisk, Pfizer, Regeneron, Roche, UCB Pharma, and Verdiva Bio, and payment or honoraria for lectures, presentations, or speakers bureaus from AbbVie, Amgen, AstraZeneca, Boehringer Ingelheim, Eli Lilly and Company, Novo Nordisk, Roche and UCB Pharma and reports a leadership or fiduciary role in HM Government, Office for Life Sciences and Chair Obesity Healthcare Programme.

## Data Availability

Lilly provides access to all individual participant data collected during the trial, after anonymization, with the exception of pharmacokinetic or genetic data. Data are available to request 6 months after the indication studied has been approved in the United States and EU and after primary publication acceptance, whichever is later. No expiration date of data requests is currently set once data are made available. Access is provided after a proposal has been approved by an independent review committee identified for this purpose and after receipt of a signed data sharing agreement. Data and documents, including the study protocol, statistical analysis plan, clinical study report, and blank or annotated case report forms, will be provided in a secure data sharing environment. For details on submitting a request, see the instructions provided at www.vivli.org.
